# Modulations of resting-static functional connectivity on insular by electroacupuncture in subjective tinnitus

**DOI:** 10.3389/fneur.2024.1373390

**Published:** 2024-03-22

**Authors:** Bixiang Zha, Yating Zhang, Feifei Shi, Ling Cheng, Zhihao Rong, Leiyu Yu, Wanting Liu, Qiuju Xue, Min Ye, Jinying Yang, Bensheng Qiu, Jun Yang

**Affiliations:** ^1^The First Affiliated Hospital of Anhui University of Chinese Medicine, Hefei, China; ^2^The First School of Clinical Medicine, Anhui University of Chinese Medicine, Hefei, China; ^3^The Second Affiliated Hospital of Anhui University of Chinese Medicine, Hefei, China; ^4^The School of Humanity and International Education and Exchange, Anhui University of Chinese Medicine, Hefei, China; ^5^Laboratory Center for Information Science, University of Science and Technology of China, Hefei, China; ^6^Medical Imaging Center, Department of Electronic Engineering and Information Science, University of Science and Technology of China, Hefei, China

**Keywords:** subjective tinnitus, electroacupuncture, rs-fMRI, functional connectivity, insular

## Abstract

**Objective:**

To explore the modulations of electroacupuncture in subjective tinnitus (ST) by comparing the difference of functional connectivity (FC) in ST patients and healthy volunteers between the insular (INS) and the whole brain region.

**Methods:**

A total of 34 ST patients were selected into electroacupuncture group (EG) and 34 age- and sex-matched normal subjects were recruited into control group (CG). The EG received acupuncture at SI19 (*Tinggong*), GB11 (*Touqiaoyin*), TE17 (*Yifeng*), GV20 (*Baihui*), GV15 (*Yamen*), GV14 (*Dazhui*), SJ13 (*Zhongzhu*), among which the points of SI19 and GB11 were connected to the electroacupuncture instrument with the density wave of 2/50 Hz, and 3 treatments per week for 10 sessions in total. The severity of tinnitus was evaluated by Tinnitus Handicap Inventory (THI), the hearing status was recorded using pure tone audiometry, and resting-state functional magnetic resonance imaging (rs-fMRI) was performed on the brain before and after treatment, the CG received no intervention yet only rs-fMRI data were collected.

**Results:**

With the electroacupuncture treatment, the total THI score, average air conduction threshold of patients of EG were significantly lower than before (*p* < 0.01), and the total effective rate was 88.24%. Compared with CG, FC of ST patients between INS and left superior temporal gyrus and right hippocampal significantly decreased before treatment, while FC of ST patients between INS and right superior frontal gyrus, left middle frontal gyrus and right anterior cuneus significantly decreased after treatment (voxel *p* < 0.001, cluster *p* < 0.05, corrected with GRF). FC of ST patients between the INS and right middle frontal gyrus, left superior frontal gyrus and right paracentral lobule showed a significant decrease after treatment (voxel *p* < 0.001, cluster *p* < 0.05, corrected with GRF). In addition, THI score in EG was negatively correlated with the reduction of FC value in INS-left superior frontal gyrus before treatment (*r* = −0.41, *p* = 0.017). Therefore, this study suggests that abnormal FC of INS may be one of the significant central mechanisms of ST patients and can be modulated by electroacupuncture.

**Discussion:**

Electroacupuncture treatment can effectively reduce or eliminate tinnitus symptoms in ST patients and improve the hearing by decreasing FC between the INS and the frontal and temporal brain regions.

## 1 Introduction

Subjective tinnitus (ST) refers to the perception of one or more abnormal sounds heard in the ear or head in the absence of an external sound source, often accompanied by symptoms such as anxiety, depression and insomnia (1). More than 740 million adults around the world have been plagued by tinnitus for a long time, and it is regarded by more than 120 million people as a major problem affecting the quality of life (2), and even suicidal tendencies (3). At present, there is no effective medication for ST (4). As an original characteristic method of the Chinese nation, acupuncture and moxibustion have been recorded to treat tinnitus as early as in the ancient book *Lingshu* (5), and can also improve tinnitus insomnia and emotional disorders (6, 7). However, acupuncture treatment of ST is still not widely recognized internationally (1), in that the modern biological mechanism of acupuncture treatment effect has not been fully interpreted.

With the development and application of neuroimaging technology, resting-state functional magnetic resonance imaging (rs-fMRI) has opened up a new perspective to study the specificity of meridian effect (8), the mechanism of acupuncture effect (9), and the influencing factors of acupuncture effect (10), etc. The impact of acupuncture on brain functional connectivity (FC) is an important trend of rs-fMRI research in the future. ST, as a typical “brain-ear interaction disorder,” lacks clear structural and organic lesions of the auditory system. The application of rs-fMRI has made certain progress in the study of central nervous mechanism of ST (11). Previous studies (12, 13) of this team showed that FC between the amygdala and the right inferior temporal gyrus and the right anterior cuneus, and between the right anterior cingulate gyrus and the left middle temporal gyrus and the superior frontal gyrus were all reduced in ST patients. Electroacupuncture treatment could reduce FC values of the amygdala, cingulate gyrus and related brain areas, promote functional recombination of auditory and emotional-related brain areas, and effectively improve clinical symptoms of ST patients. Recent studies suggested that insula (INS) was the core region of the neural pathway composed of the auditory cortex, the insula and the parahippocampus/posterior cingulate gyrus, and abnormal FC between INS and the auditory cortex, the posterior cingulate gyrus and the parahippocampus existed in ST patients (14). And whether the central action mechanism of acupuncture treatment of ST is related to the regulation of functional connections between INS and related brain regions. Therefore, on the basis of confirming the clinical efficacy of electroacupuncture for ST, this study applied the FC analysis method of rs-fMRI to explore the changes of INS network function in ST patients, and tried to explore the image markers of electroacupuncture for ST.

## 2 Methods

### 2.1 Source and grouping of research objects

According to Hayasaka’s study (15) on sample size analysis of fMRI data, stable brain responses could be observed in at least 12 cases. Therefore, the electroacupuncture group (EG) in this study consisted of 34 ST patients who were treated in the outpatient department of the Second Department of Acupuncture and Rehabilitation of Anhui Provincial Hospital of Traditional Chinese Medicine from March 2021 to May 2023, and the control group (CG) consisted of 34 healthy volunteers who were publicly recruited and matched with the age and gender of the EG. This study was approved by the Ethics Committee of Anhui Provincial Hospital of Traditional Chinese Medicine (Ethics number: 2021AH-29).

### 2.2 Diagnostic criteria

According to the 2020 *European Multidisciplinary Tinnitus Guidelines: Diagnosis, Assessment and Treatment* (4), the first complaint from patients is the self-awareness of ringing in the ear without external sound sources, and the nature of the sound is cicada chirping, buzzing, hissing, wind blowing, etc.; unilateral or bilateral, continuous or intermittent, accompanied by hearing loss, insomnia, vertigo, anxiety; physical examination: auricle, external ear canal no deformity, tympanic membrane intact. Patients must meet the above criteria to be diagnosed as ST and have a professional otolaryngologist to assist in the diagnosis.

### 2.3 Inclusion criteria

Participants ① met the diagnostic criteria of ST; ② were between the ages of 18 and 65, right-handed; ③ suffered from subjective persistent or intermittent tinnitus, and had received no acupuncture treatment in the last 3 months; ④ were of an illness grading criteria THI second to fifth grade; ⑤ suffered no obvious abnormalities in the intracranial and ear canals by conventional MRI scan; ⑥ met the requirements of magnetic resonance examination (no metal dentures, pacemakers, stents and no claustrophobia, etc.); ⑦ volunteered to participate in this study and signed the informed consent.

### 2.4 Exclusion criteria

The exclusion criteria were those who ① possessed a history of mental or nervous system diseases; ② had a clear past record of organic diseases, such as heart, liver, kidney and hematopoietic system diseases; ③ were pregnant and lactating women; ④ might not be able to cooperate in the treatment, and could not independently complete the scale and follow-up.

### 2.5 Treatment plan

**Figure 1:**
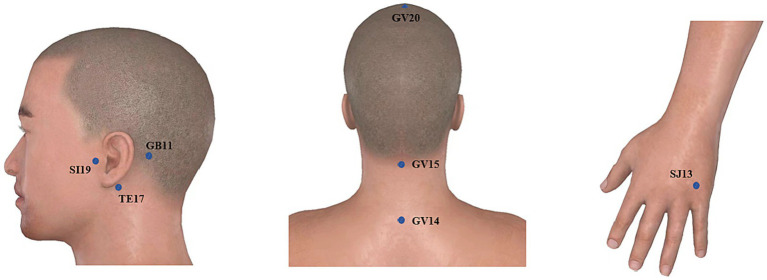
Acupoints selected for acupuncture treatment of ST.

In this study, based on the clinical treatment experience of ST of Professor Yang Jun, the National Celebrated Traditional Chinese Medicine Expert in China, with the main treatment principle of “Ningshen Tongqiao (Calming the mind and clearing the orifices), Tongjing Huoluo (Expediting channel and activating meridian),” the acupoints on the affected side of SI19 (*Tinggong*), GB11 (*Touqiaoyin*), TE17 (*Yifeng*), SJ13 (*Zhongzhu*) and the acupoints on the governor vessel of GV20 (*Baihui*), GV15 (*Yamen*), GV14 (*Dazhui*) were selected (Figure 1). The specific locations of acupoints were referred to the national standard of the People’s Republic of China: *Name and Location of Meridian Points* (GB/T 12346-2021).

Operation methods: 75% medical alcohol was used to disinfect the acupuncture site, and the disposable sterile acupuncture needles’ size was 0.35 × 40 mm. For each participant in a sitting position, SI19 was, with an open mouth, needled to a depth of 30–40 mm with the needle tip toward the inner ear until the ear had a slight electrical shock sensation without twisting the needle; GV15 was needled to a depth of 25 mm–30 mm with the needle tip toward the lower jaw with the mouth closed, the nose pinched and air blown until the patient felt local pain and distension; GB11, and TE17 were obliquely punctured to a depth of 20 mm–30 mm with the needle tip toward the ear until the ear had a slight electrical shock sensation; GV20 and SJ13 were obliquely needled to a depth of 20 mm until achieving *de qi* needling sensation (a composite of sensations including soreness, numbness, distention, heaviness, and other sensations), which is believed to be an essential component of acupuncture efficacy; GV14 was obliquely upward needled to a depth of 20 mm–30 mm with needle sensation radiating around. After the completion of acupuncture, the points of SI19 and GB11 were connected to the electroacupuncture instrument with the density wave of 2/50 Hz. The current intensity was determined by the patient’s tolerance, and theretention time of needles was 30 min. The acupuncture was performed by Professor Yang in the acupuncture department of Anhui Provincial Hospital of Traditional Chinese Medicine. Acupuncture treatment was performed 3 times a week, once every other day, and the electroacupuncture group received acupuncture treatment 10 times, and if it was not complete, the treatment was stopped.

### 2.6 Observation indicators

The score of THI (4) was mainly assessed from three aspects: severity, functionality and emotion, and scored according to 4-point narrative scales: “yes” (4 points), “sometimes” (2 points) and “none” (0 points). A total of 100 points are for 25 items which were divided into the first level (0–16), the second level (18–36), the third level (38–56), the fourth level (58–76), and the fifth level (78–100). The score decreased by ≥7 points before and after treatment was regarded as effective treatment, ≥20 points was regarded as obvious effect, and the disappearance of tinnitus symptoms was regarded as recovery (16).

Pure tone hearing threshold of 250–8,000 Hz was examined with a clinical diagnostic electrical audiometer (MI44, Maico) to calculate the average air conduction hearing threshold. A lower average hearing threshold means better hearing.

rs-fMRI data were acquired using GE’s 8-channel skull coil 3.0 T scanner. EG underwent fMRI scan before and 2 days after treatment, respectively, and CG underwent fMRI scan after inclusion. Before scanning, all metal and magnetic objects on the body were removed, and noise-reducing earplugs and head-fixing sponge pads were prepared for the subjects. The subjects remained relaxed, calm, awake and not thinking with their eyes closed during scanning. Structure images were obtained using 3D T1 BRAVO sequence: repetition time (TR) = 8.2 ms, echo time (TE) = 3.2 ms, inversion time = 450 ms, turn angle = 12°, scan field = 256 mm × 256 mm, matrix = 256 × 256, layer thickness = 1.0 mm, voxel = 1 mm × 1 mm × 1 mm, number of layers = 188, total duration = 4 min 43 s. Gradient echo plane echo imaging sequence was used to collect resting-state fMRI: TR = 2,400 ms, TE = 30 ms, turning angle = 90, scanning field = 192 mm × 192 mm, matrix = 64 × 64, layer thickness = 3.0 mm, voxel = 3 mm × 3 mm × 3 mm, number of layers = 46, 217 time points in total, total duration = 8 min 40 s.

### 2.7 Data processing

#### 2.7.1 Clinical data analysis

SPSS 25.0 software was used for statistical analysis of clinical scale data, and two-sample *t*-test and Chi-square test were conducted to compare the matching degree of general information (age and gender) of the study subjects. The THI scores and average hearing threshold scores of ST patients before and after acupuncture were tested for normality and homogeneity of variance. If the normal distribution was met, the paired *T*-test was used to compare whether there were differences in THI and average hearing threshold before and after acupuncture; if not, the rank sum test was used.

#### 2.7.2 rs-fMRI data preprocessing

The software package DPABI (17) was used to preprocess imaging data according to the following processes: ① Format conversion: the original data was changed from DICOM format to NIFTI format, and the data were checked one by one to see if there were artifacts or large head movements; ② Time correction: the first 10 time points were removed; ③ Head motion correction: data with head motion exceeding 3 mm or 3° were excluded; ④ Spatial standardization: data were co-registered with the structural images, spatial normalized to MNI template, and voxels re-sampled to 3 mm × 3 mm × 3 mm resolution; ⑤ Regression interference factors: head movement and other signals, white matter and cerebrospinal fluid in the analyzed data were used as covariate regression; ⑥ Spatial smoothing: half-height full-width Gaussian kernel of 6 mm × 6 mm × 6 mm was used to smooth the image; ⑦ Filter: 0.01–0.08 Hz low-band signal was kept.

#### 2.7.3 FC calculation and analysis of rs-fMRI data

Using WFU PickAtlas software,^1^ double-sided INS was taken as region of interest (ROI). DPABI software was used to calculate ROI and FC between the whole brain. For each participant, the Pearson correlation coefficient between the mean time series of ROI and the time series of every voxel across the whole brain was calculated, and the coefficients were further converted to a *z*-value using a Fisher *r*- to- *z* transformation to improve the normality.

DPABI was used to conduct two independent samples *t*-test for FC data of the before-electroacupuncture treatment in ST patients and the CG, two independent samples *t*-test for FC data of the after-electroacupuncture treatment in ST patients and the EG, and paired *t*-test for FC data of the after- and before-electroacupuncture treatment in ST patients. The final results were corrected using Gaussian random fields (GRF), with voxel level *p* < 0.001 and cluster level *p* < 0.05.

SPSS 25.0 was used to analyze the correlation between THI scores of ST patients and results with differences within the group, and Pearson correlation coefficient was calculated.

## 3 Results

### 3.1 Clinical general data

A total of 34 ST patients and 34 age- and sex-matched normal subjects participated in and completed the study. Their characteristics are summarized in Table 1.

**Table 1:** Demographic characteristics of the study participants (^−^*x* ± *s*).

Groups	Number	Age	Gender	
			Male	Female
Electroacupuncture group	34	46.91.68 ± 10.48	15	19
Control group	34	46.82 ± 12.24*	16	18

Compared with electroacupuncture group *p > 0.05.

### 3.2 Clinical data of ST patients before and after electroacupuncture treatment

**Figure 2:**
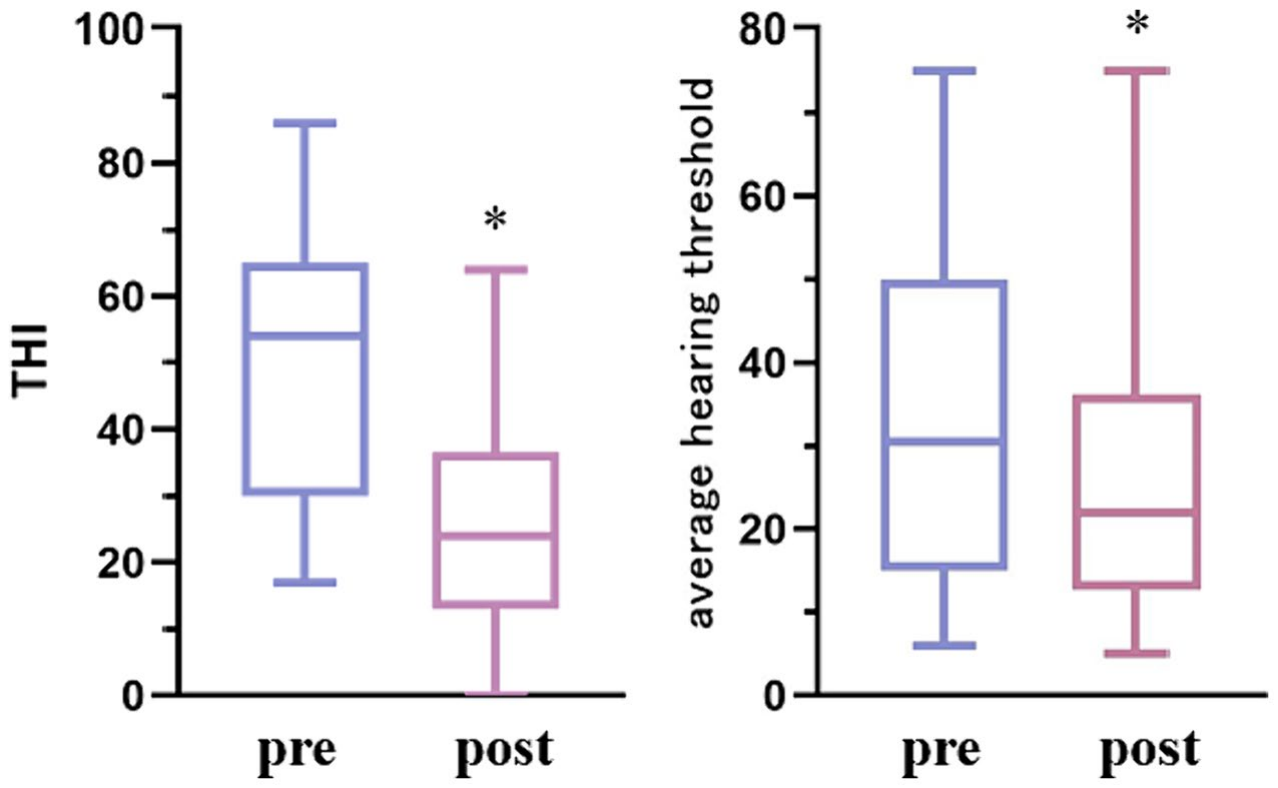
Comparison of THI and mean auditory threshold of ST patients before and after electroacupuncture treatment. Compared with pre-treatment *p < 0.05.

Compared with pre-treatment, THI and average hearing threshold scores of ST patients in the EG significantly decreased post-treatment (*p* < 0.05), as shown in Table 2 and Figure 2.

**Table 2:** Comparison of THI and average hearing threshold scores in ST patients before and after electroacupuncture treatment (^−^*x* ± *s*).

Groups	Number	THI scores	Average hearing threshold scores/dB HL
Pre-treatment	34	50.56 ± 19.91	33.74 ± 19.19
Post-treatment	34	25.94 ± 18.18*	25.21 ± 16.38*

Compared with pre-treatment *p < 0.05.

THI score was used to evaluate the effective rate of ST patients treated with electroacupuncture. Among the 34 ST patients treated with acupuncture, 2 cases were cured, 16 cases were significantly effective, 12 cases were effective, and 4 cases were ineffective, with a total effective rate of 88.24%.

### 3.3 Comparison of fMRI data between ST patients and CG

**Figure 3:**
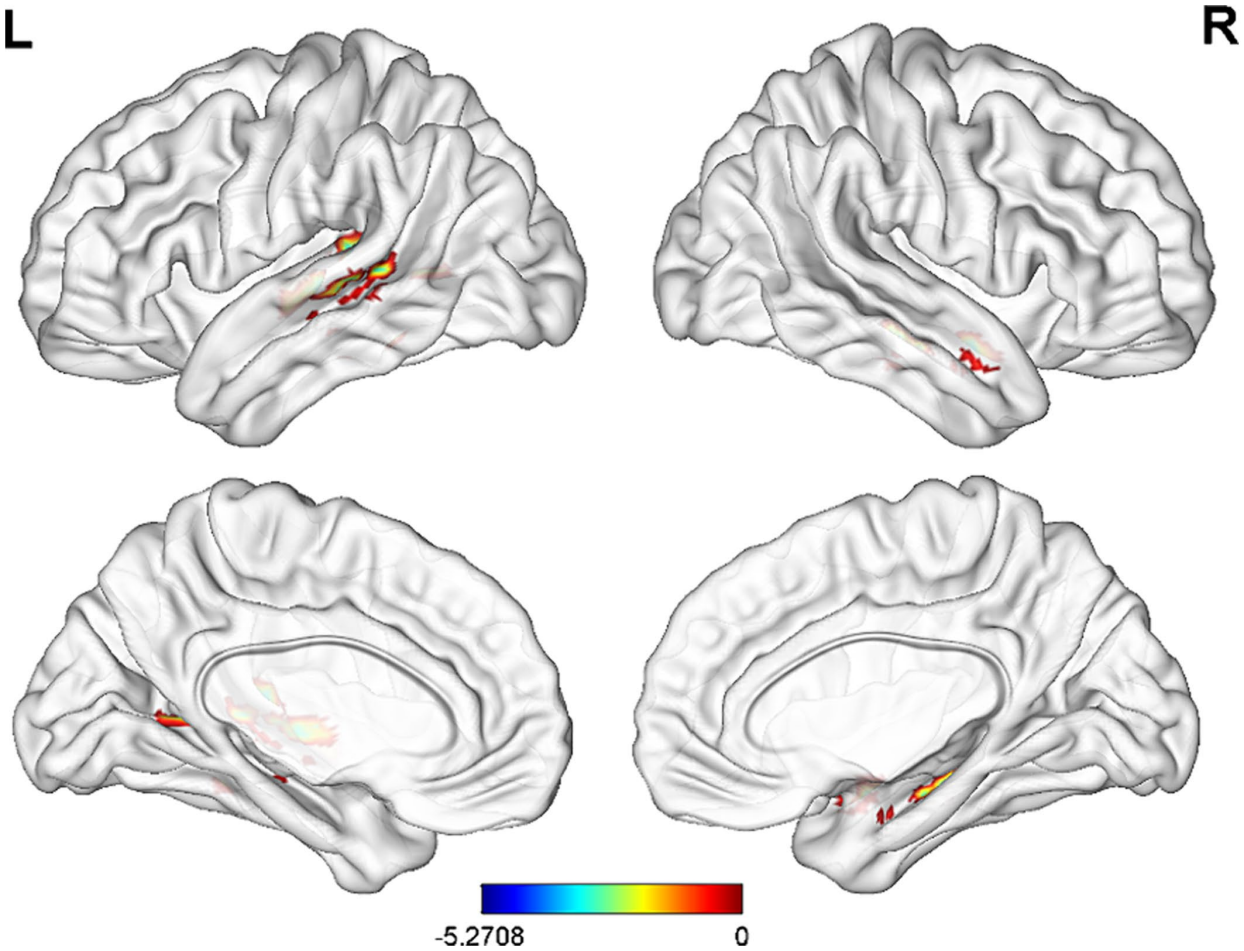
Significant differences in FC between before electroacupuncture treatment in ST patients and CG. The result was corrected for GRF with voxel *p* < 0.001, cluster *p* < 0.05.

**Figure 4:**
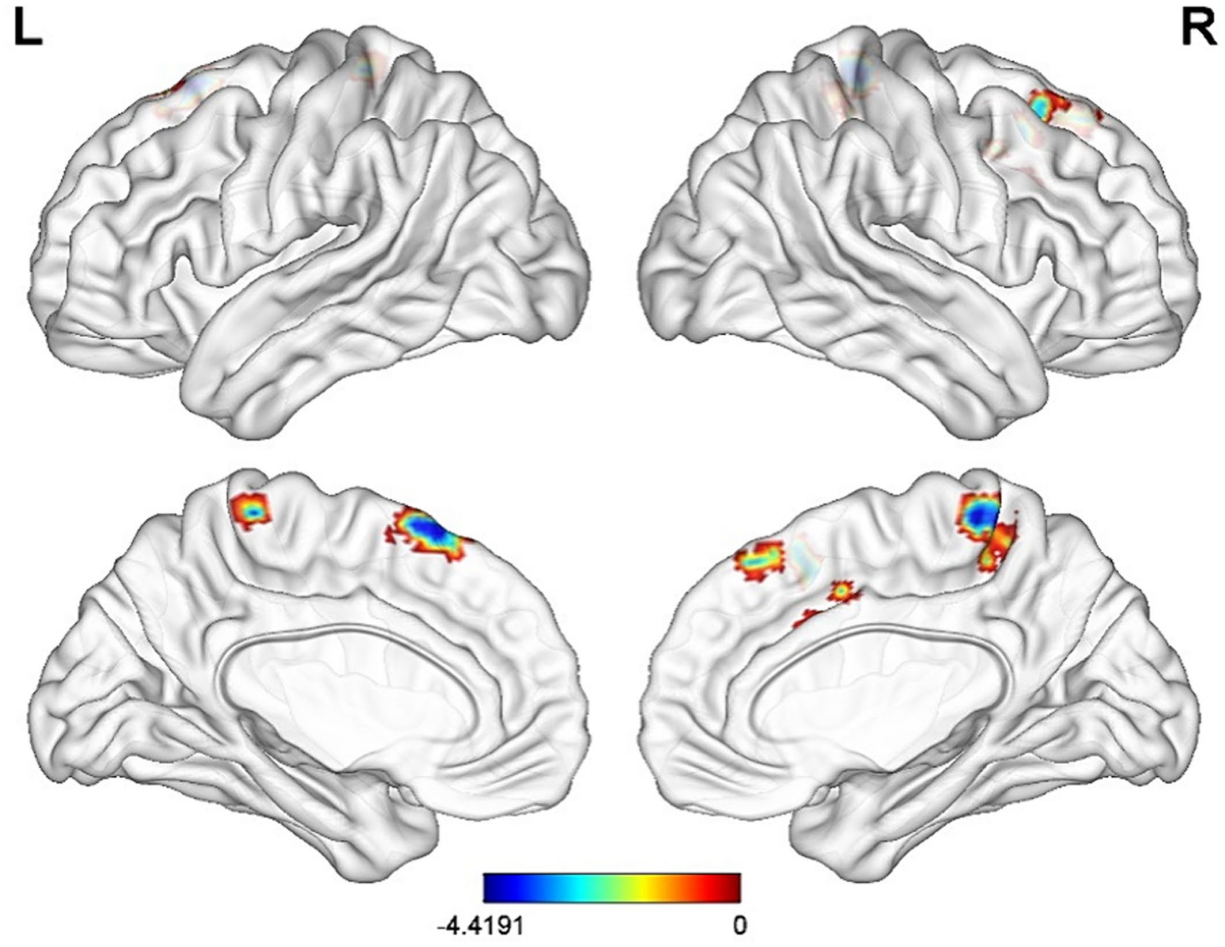
Significant differences in FC between after electroacupuncture treatment in ST patients and CG. The result was corrected for GRF with voxel *p* < 0.001, cluster *p* < 0.05.

**Figure 5:**
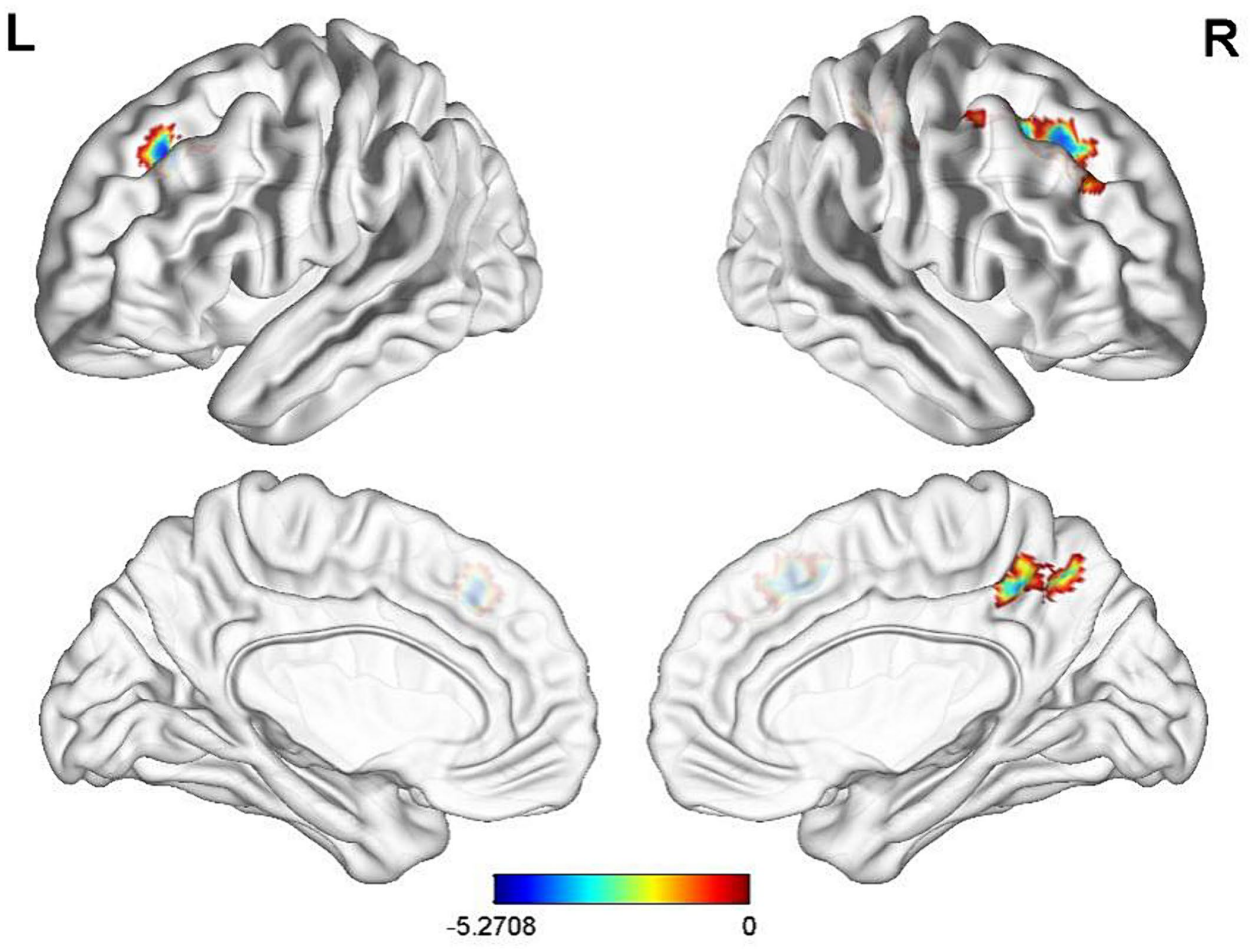
Significant differences in FC of the INS between before-and after-electroacupuncture treatment in ST patients. The result was corrected for GRF with voxel *p* < 0.001, cluster *p* < 0.05.

Compared with CG, FC of before electroacupuncture treatment in ST patients between INS and left superior temporal gyrus and right hippocampal significantly decreased before electroacupuncture (Table 3 and Figure 3, voxel *p* < 0.001, cluster *p* < 0.05, corrected with GRF), while FC of ST patients between INS and right superior frontal gyrus, left middle frontal gyrus and right anterior cuneus significantly decreased after treatment (Table 4 and Figure 4, voxel *p* < 0.001, cluster *p* < 0.05, corrected with GRF). FC of ST patients between the INS and right middle frontal gyrus, left superior frontal gyrus and right paracentral lobule showed a significant decrease after treatment (Table 5 and Figure 5, voxel *p* < 0.001, cluster *p* < 0.05, corrected with GRF).

**Table 3:** Comparison of FC in the INS between before electroacupuncture treatment in ST patients and CG.

Regions	Voxels	MNI			*T*-value (Peak intensity)
		X	Y	Z	
Left superior temporal gyrus	192	−42	−33	0	−4.611
Right hippocampus	102	45	3	−21	−4.559

The result was corrected for GRF with voxel *p* < 0.001, cluster *p* < 0.05. MNI, Montreal Neurological Institute. A negative *T*-value indicates a decreased FC.

**Table 4:** Comparison of FC in the INS between after electroacupuncture treatment in ST patients and CG.

Regions	Voxels	MNI			*T*-value (Peak intensity)
		X	Y	X	
Right superior frontal gyrus	113	21	18	42	−5.252
Left middle frontal gyrus	42	−24	30	42	−5.270
Right anterior cuneus	32	3	−51	45	−4.515

The result was corrected for GRF with voxel *p* < 0.001, cluster *p* < 0.05. MNI, Montreal Neurological Institute. A negative *T*-value indicates a decreased FC.

**Table 5:** Comparison of FC in the INS between before and after electroacupuncture treatment in ST patients.

Regions	Voxels	MNI			*T*-value (Peak intensity)
		X	Y	X	
Right middle frontal gyrus	67	12	15	30	−4.419
Left superior frontal gyrus	66	−6	21	60	−4.324
Right paracentral lobule	56	3	−36	63	−4.388

The result was corrected for GRF with voxel *p* < 0.001, cluster *p* < 0.05. MNI, Montreal Neurological Institute. A negative *T*-value indicates a decreased FC.

### 3.4 Correlation analysis between THI score and FC value

**Figure 6:**
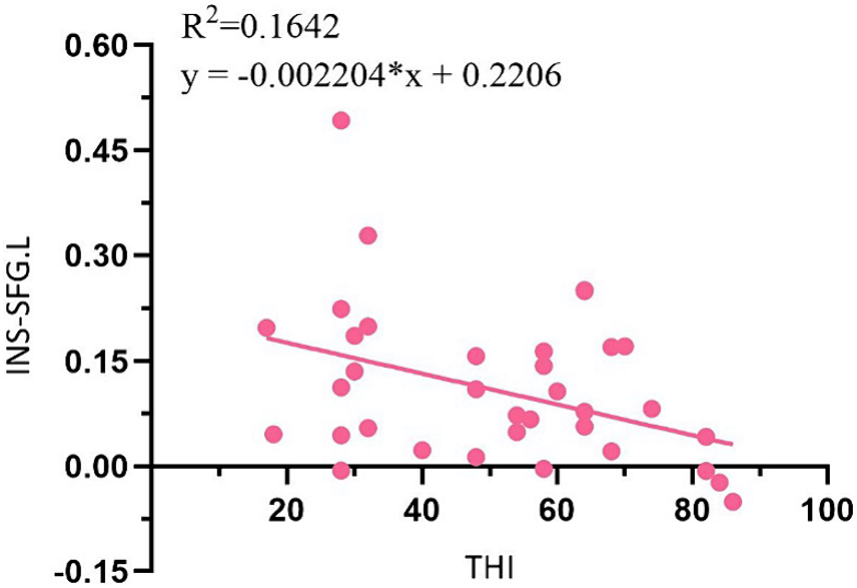
Correlation analysis between THI score and FC value in ST patients. SFG, superior frontal gyrus; L, left.

In addition, THI score in EG was negatively correlated with the reduction of FC value in INS-left superior frontal gyrus before electroacupuncture treatment (Figure 6, *r* = −0.41, *p* = 0.017). Unfortunately, no similar results were found after electroacupuncture treatment.

## 4 Discussion

ST is one of the three diseases in otolaryngology difficult to be cured, and its pathogenesis may be related to one or more factors such as inner ear blood supply disorder, viral infection, autoimmune disease and emotional disorder (18–20). This study puts forward the principle and characteristic treatment of “NingshenTongqiao, TongjingHuoluo” electroacupuncture for ST proposed by Professor Yang, which is based on the possible pathogenic factors of ST “qi and blood stagnation, spirit and auditive orifice blockage” in TCM and “insufficient blood supply, nerve damage, immune inflammation” in modern biomedicine (21, 22). The clinical results showed that electroacupuncture could reduce THI and average hearing threshold scores, improve tinnitus symptoms and hearing status of ST patients with the total effective rate 88.24%, which might provide clinical evidence for the study of brain effect mechanism of electroacupuncture treatment of ST patients.

The INS cortex is located deep in the lateral sulcus of the brain and known as the limbic integrated cortex, which integrates various functions such as auditory processing, cognition and social-emotional processing (23). Researchers use auditory stimuli to activate specific INS, which are key brain regions for recognizing and processing attitudes (negative or positive) to sound stress (24). Cortical excitatory toxicity of INS can maintain hearing sensitivity while producing tinnitus (25). Through neuroimaging technology, many researchers found that INS in ST patients showed excitability or inhibition, and explored it as a candidate region for the final common pathway of ST (26). However, there were great differences in the studies on its related FC in the literature reports, and even opposite results (27, 28).

In this study, the FC analysis method of rs-fMRI showed that the FC values between INS and left superior temporal gyrus and the right hippocampus were decreased in ST patients. The superior temporal gyrus contains the non-primary auditory cortex, which exhibits complex auditory encoding in its local cortical location and unique acousto-acoustic and prosodic features, making it a key site for sound processing (29). Studies have shown that the FC value of the temporal lobe brain network in ST patients is weakened (30), especially between the superior temporal gyrus and non-auditory brain areas such as amygdala, nucleus accumbens, cerebellum and posterior central gyrus (31). In addition, studies have found that both the metabolism of superior temporal gyrus and the volume of gray matter of INS decreased (32). The hippocampus belongs to the limbic system of the brain, which is closely related to higher neural activities such as memory, learning and cognition, and plays a key role in ST pathology (33). Studies have shown that the hippocampus is impaired in auditory disorders such as tinnitus and hearing loss, and the persistence of symptoms is related to memory and cognitive mechanisms (34). Therefore, the results of this study suggest that tinnitus severity may be correlated with sound perception, memory and cognitive function.

In recent years, rs-fMRI technology has been used to explore the mechanism of acupuncture regulation of brain network and has obtained great research results, but relatively few studies have been conducted on the acupuncture effect of ST. Studies have shown that acupuncture can increase the concentration of oxyhemoglobin in temporal lobe and promote the activation of auditory cortex in tinnitus patients (35). There are also studies that use auricular point stimulation to promote blood oxygenation-dependent signal changes in subjects’ prefrontal, auditory, and limbic cortex (36). This study showed that FC of ST patients between the INS and right middle frontal gyrus, left superior frontal gyrus and right paracentral lobule showed a significant decrease after electroacupuncture treatment. Frontal lobe is an important region involved in tasks such as memory, resting state and cognitive control (37). Changes in INS and orbitofrontal cortex *α*1 frequency band power were positively correlated with changes in tinnitus loudness and percentage of distress scored on the tinnitus sensory Numerical Rating Scale (38). The tinnitus awareness and emotional response of ST patients are mainly reflected in the structural changes of the frontal and precuneus lobes (39). Previous studies by this team also showed that FC values of ST patients between anterior cingulate gyrus and medial frontal gyrus, superior frontal gyrus and thalamus decreased after electroacupuncture compared with before electroacupuncture. And the same result were founded between the amygdala and the right superior frontal gyrus and the left cingulate gyrus. The INS, anterior cingulate gyrus and amygdala are the main nodes of the salience network. Therefore, it can be speculated that electroacupuncture may treat ST by regulating the salience network and functional remodeling of auditory and emotional, cognitive related brain areas. However, electroacupuncture treatment may not fully return ST patients to normal state. Compared with CG, the FC values of ST patients in EG between INS and right superior frontal gyrus, left middle frontal gyrus and right anterior cuneus decreased after electroacupuncture treatment. While alleviating the loudness of tinnitus and the emotions of patients, most patients still had the perception of tinnitus, which was consistent with this total effective rate of 88.24%. In addition, THI score in EG was negatively correlated with the reduction of FC value in INS-left superior frontal gyrus before treatment. Unfortunately, perhaps due to insufficient electroacupuncture treatment cycles, no relevant results were found after treatment. However, THI score and FC value of superior frontal gyrus decreased significantly after electroacupuncture treatment. This result, to a certain extent, may suggest that there is a substantial change in frontal gyrus behind the improvement of symptoms in ST patients, possibly due to the moderating effect of acupuncture on emotions. It is worth further research.

In summary, this study showed that there were abnormal FC changes in ST patients between INS and auditory cortex and brain regions related to emotion and cognition. Electroacupuncture therapy can improve the clinical symptoms of ST by regulating INS and functional remodeling of related brain areas. Combined with previous studies, it can be speculated that the prominence network with INS, anterior cingulate gyrus and amygdala as the main nodes may be the core network of the brain effect of electroacupuncture, which may provide a visual basis for the research and clinical promotion of image markers of electroacupuncture treatment of ST. However, this study still has some limitations. For example, the clinical features of ST patients recruited were not detailed enough to distinguish whether INS and FC in related brain areas were different in different clinical features. In the future, more stringent screening inclusion criteria will be developed to study the brain functional network of ST patients, such as in studying different stages, with or without hearing loss, and with laterality of tinnitus. In addition, this study only conducted a comparison before and after electroacupuncture treatment, and the treatment cycle was short. In the later stage, more convincing clinical studies will be carried out in combination with randomized controlled trials to provide more objective evidence-based data for the study of the mechanism of electroacupuncture ST.

## 5 Conclusion

Electroacupuncture treatment of ST can alleviate or eliminate the symptoms of tinnitus, improve the level of hearing and improve the quality of life. It is a potential research direction to study the recombination mechanism of brain network in the treatment of ST by electroacupuncture through rs-fMRI.

## Data availability statement

The original contributions presented in the study are included in the article/supplementary material, further inquiries can be directed to the corresponding author.

## Ethics statement

The studies involving humans were approved by the Ethics Committee of Anhui Provincial Hospital of Traditional Chinese Medicine. The studies were conducted in accordance with the local legislation and institutional requirements. The participants provided their written informed consent to participate in this study.

## Author contributions

BZ: Writing – original draft, Supervision, Writing – review & editing, Conceptualization, Project administration. YZ: Writing – original draft, Formal analysis, Writing – review & editing, Data curation, Conceptualization. FS: Writing – review & editing. LC: Writing – review & editing, Investigation. ZR: Data curation, Software, Writing – original draft. LY: Data curation, Writing – review & editing. WL: Data curation, Writing – original draft. QX: Writing – original draft, Project administration. MY: Writing – review & editing. JiY: Software, Writing – review & editing. BQ: Software, Writing – review & editing. JuY: Methodology, Project administration, Writing – original draft, Writing – review & editing, Conceptualization, Funding acquisition.

## Footnotes

^1^ https://www.wfubmc.edu
